# miR-142-3p suppresses apoptosis in spinal cord-injured rats

**DOI:** 10.1515/tnsci-2020-0105

**Published:** 2020-05-18

**Authors:** Jun Zheng, Jing Kuang, Xianyu Zhang, Daya Luo, Weijing Liao

**Affiliations:** Department of Neurorehabilitation, Zhongnan Hospital of Wuhan University, Wuhan, Hubei, 430071, China; Department of Plastic Surgery, Jinan Central Hospital Affiliated to Shandong University, Jinan, Shandong, 250013, China; Orthopedics Department, ShangRao People’s Hospital, Shangrao, Jiangxi, 334000, China; Department of Biochemistry and Molecular Biology, School of Basic Medical Sciences, Nanchang University, Nanchang, Jiangxi, 330006, China

**Keywords:** Bax, caspase-3, caspase-9, miR-142-3p, spinal cord injury

## Abstract

**Introduction:**

Spinal cord injury (SCI) leads to abnormal expression of miRs, leading to secondary responses such as oxidative stress, inflammation and apoptosis. In the present work, we screened the miRs involved and the associated pathway.

**Methods:**

In a rat model of SCI, the microarray analysis for expression of miRs at various time points post-SCI was done. The locomotor analysis was done by Basso, Beattie and Bresnahan score, and Cresyl violet staining was done for lesion volume and TUNEL assay was done for apoptosis in neuronal cells. The expression of apoptotic proteins was done by the western blot study.

**Results:**

It was evidenced that the expression of the number of miRs was altered on the 14th day post-SCI, and miR-142-3p was found to be the most significantly suppressed miR. The results suggested that overexpression of miR-142-3p by its agomir-attenuated functional recovery decreased lesion size and apoptosis of neuronal cells in rats subjected to SCI. The luciferase assay indicated that miR-142-3p blocked the levels of Bax, which is a significant activator of the mitochondrial apoptotic pathway (MAP) via targeting the 3′UTR region of BV-2 cells, and in addition, pc-DNA-Bax restored Bax and inhibited the correcting role of miR-142-3p in hydrogen peroxide-treated BV-2 cells. The findings suggested that miR-142-3p may inhibit the MAP by inhibiting the expression of cleaved-caspase-3/-9 and Bax in SCI rats.

**Conclusion:**

This study concludes that miR-142-3p may attenuate the functional recovery and decrease apoptosis in neuronal cells via inhibiting the MAP in the spinal cord-injured rats, confirming miR-142-3p as a potential therapeutic target in treating SCI.

## 1 Introduction

Spinal cord injury (SCI) is one of the common injuries of the spine causing permanent disability such as paralysis, loss of sensitivity and loss of movement [1]. Every year, a large number of patients are reported to be affected with SCI [2]. Currently, SCI has been the biggest challenge for neuro physicians [3]. Apoptosis plays a crucial role in functional and physical deficits in SCI, and it is a major hurdle in treating SCI [4,5]. Therefore, developments of new therapeutic approaches, which can target and suppress apoptosis for countering SCI, are needed. Apoptosis, which is also defined as programmed cell death, is a key feature in neuronal tissue damage after SCI [6]. In the previous study, two pathways, namely, the mitochondrial and death receptor pathways, have been identified to play an important role in inducing apoptosis [7]. In the mitochondrial pathway, B-cell lymphoma-2 also called Bcl-2 is a group of genes that regulate apoptosis via antiapoptotic proteins (Bcl-2) and proapoptotic proteins (Bcl-2 homologous, Bax and Bcl-2 agonist) [8]. The collapse of the mitochondrial membrane potential is identified as the important process in the mitochondrial apoptosis cascade, which leads to the transfer of cytochrome *C* (CYT-*C*) from the mitochondria to the cytosol [9]. Then, CYT-*C* along with dATP and apoptotic protease-activating factor 1 (Apaf-1) in cytosol leads to the cleavage of caspase-9 and p-caspase-9 [10]. Caspase-9 further leads to the cleavage of caspase-7, caspase-6 and caspase-3 [11]. Hence, the release of CYT-*C* is a very important step for the activation of p-caspase-9 in the apoptosis-induced cell death.

MiRNAs (miRs) comprise 21–23 nucleotides, and they can modulate genes at the posttranscriptional level via suppressing translation or inducing degradation [12,13]. miRs have been found to regulate at least 65% of genes in the human genome [14]. So far studies have identified multiple miRs in the spinal cord and the brain, which are believed to regulate functioning and plasticity [15,17]. In addition, miRs have been identified to play a key role in the development of the nervous system and may be crucial in the process of cell differentiation in specific organs [16]. Earlier studies have demonstrated that the spinal injury may lead to abnormal expression of miRs, which is a response against a number of secondary injuries, including oxidative stress, apoptosis and inflammation; this also regulates the expression of some specific genes [18,19]. Recently, growing evidence suggest the involvement of a number of miRs in regulating apoptosis involving mitochondrial cascade in various disorders [20,21,22]. Hence, in the present work, we speculated that the spinal injury-mediated miRs may encourage apoptosis by activating the mitochondrial apoptosis pathway. Here, we developed a rat model of the SCI followed by the microarray analysis for identifying the expression profiles of miRs in injured spinal tissues. Subsequently, we evaluated the role of miR-142-3p in the SCI-mediated apoptosis and the mechanisms involved.

## 2 Material and methods

### 2.1 Cell lines and treatment

In this work, we used immortalized murine BV2 cell lines provided by the Zhongnan Hospital of Wuhan University, China, and were incubated in the modified DMEM media (ThermoFisher, USA). The medium was supplemented with fetal bovine serum (10%) and 100 U/mL each of streptomycin and penicillin (10%) and maintained at 37℃ under humid conditions with 5% CO_2_. The BV2 cell lines were exposed to various concentrations (50, 100, 200 and 400 µM) of H_2_O_2_ (30%; Merck, USA) for 10 h for the process of inducing cell injury.

### 2.2 Animals and treatment

We used Sprague-Dawley rats aged between 6 and 7 weeks (*n* = 36 rats) weighing approximately between 225 and 250 g. The animal study protocols were sanctioned by the Animal Ethical Review Board of Zhongnan Hospital of Wuhan University, China. The rats were housed under pathogen-free standard lab conditions at 25℃ with a relative humidity of 50–60% in 12 h dark and light cycle. The rats were provided free access to food and water. The rats were divided into four groups (*n* = 6/group), namely, the SCI-induced group, sham-operated group, miR-142-3p agomir group and miR-142-3p antagomir group (NC group). The sham-operated rats were subjected to laminectomy at T10 without the weight-drop injury; similarly, the SCI group rats underwent T10 laminectomy by the New York spinal segment impactor, and miR-142-3p antagomir-(NC) and agomir-treated rats were subjected to SCI and were given dose intrathecally (1 µL/h, 20  nmol/mL). The agomir and antagomir were obtained from RiboBio (Guangzhou, China).


**Ethical approval:** The research related to animals use has been complied with all the relevant national regulations and Animal Ethical Review Board of Zhongnan Hospital of Wuhan University, China, for the care and use of animals.

### 2.3 Establishment of SCI model

For inducing SCI, the rats were subjected to anesthesia by intraperitoneal injection of 50 mg/kg sodium pentobarbital followed by laminectomy at the T9–T10 by opening the cord below without disturbing the dura. Clamping of T8 and T11 disc was done for stabilizing the spinal cord, and then, the dorsal side of the cord was submitted to weight drop injury as described in the New York impactor process [23]. In sham rats, the laminectomy of T10 was performed by removing the weights.

### 2.4 miRNA microarray analysis

For studying the expression of miRs in the spinal tissue of rats, we sacrificed rats with the pentobarbital injection (*n* = 2 from each group). From each animal, the spinal cord tissue segment (10 mm) along with the injury epicenter was isolated and freezed in liquid nitrogen. The total RNA was separated from the spinal tissue with the help of TRIzol reagent (ThermoFisher, USA) following the supplied instructions, and the RNAs were purified using the RNeasy cleanup kit (Qiagen, Germany). The absorbance was measured using Shimadzu 1800 spectrophotometer (Shimadzu). The miRs were isolated using the microarray labeling kit (ThermoFisher, USA) and were then hybridized using the Exiqon miRCURY LNA array (Biocompare, USA). The slides were scanned using the SureScan microarray scanner (Agilent, USA). The miRs having an intensity of more than 50 were selected for further study, and the expression of miRs was normalized by median normalization. The miRs were evaluated by using volcano plots. The final clustering was utilized for determining the variations in the expression profiles of miRs.

### 2.5 Isolation of RNA and RT-PCR

The total RNA was isolated from the spinal tissue segment at the injured site with the help of TRIzol reagent following the supplied instructions. The reverse transcription was carried out using the TaqMan miR reverse transcription kit in accordance to supplied instructions. The PCR was done with the help of the TaqMan miR assay kit (ThermoFisher, USA) on a real-time PCR system (ThermoFisher, USA) following the manufacturer’s instructions. The primers used in the study were miR-142-3p, 5′-GTCGTATCCAGTGCAGGGTCCGAGGTATTCGCACTGGATACGACTCCATA-3′, and Bax, 5′-GGCTGGACACTGGACTTCCT-3′, U6, 5′-CTCGCTTCGGCAGCACA-3′. The expression of miR was observed relative to U6. All the experiments were done in triplicate.

### 2.6 Behavioral study

The impact of SCI on behavioral parameters was evaluated by the Basso, Beattie and Bresnahan (BBB) scoring system for locomotor activity done on the 1st, 7th, 14th and the 28th day after SCI [26]. The experiment evaluated the locomotor activity for 4 min. The evaluation was done by a trained person. We selected the open field test for locomotion coupled with BBB scoring as described earlier [26]. For minimizing the differences in the study, the experiments were performed by two independent investigators, and the mean was recorded.

### 2.7 Evaluation of lesion volume

To mark the lesion volume due to SCI or defined treatments (antagomir and agomiR), the animals were anesthetized by injecting pentobarbital (50 mg/kg). The rats were perfused with isotonic saline solution (0.9%, 250 mL) and then by the mixture of 4% paraformaldehyde in 0.1 M phosphate-buffered saline for 20 min. Then, the spinal segment measuring 1 cm at the injured site was harvested and fixed in the mixture of 4% paraformaldehyde in 0.1 M phosphate-buffered saline for 12 h. The tissues were then fixed in paraffin, and transverse sections having a thickness of 10 µm were produced using a microtome. The sections were mounted on slides. These sections of spinal tissue at the injured site were subjected to staining with Cresyl-violet acetate (0.5%) for 45 min at 37°C and viewed under a microscope at 200× magnification. The images of sections were processed to outline the lesion part and the spared areas with the help of the image analysis software. The spared tissues were regarded as the remaining area with no injured part with normal features. The portion of the section with minimal spared tissue was regarded as the injury epicenter.

### 2.8 Terminal deoxynucleotidyl-transferase-mediated dUTP nick-end labeling (TUNEL) assay

To study the extent of apoptosis in the spinal tissues, we subjected the tissues sections for TUNEL staining. The study was performed using the TUNEL apoptosis assay kit (Biovision, USA). The TUNEL staining showed the apoptotic cells in the spinal tissues, following the supplied instructions. Briefly, the sections were immersed in the TUNEL reagent for 45 min at room temperature, the nuclei were stained with DAPI (1 µg/mL) for 10 min. The sections were quantified for a number of DAPI-positive cells and observed in 15 random areas form each slide with the help of a fluorescent microscope.

### 2.9 Luciferase reporter assay

We performed bioinformatics analysis by TargetScan for finding the favorable binding site between miR-142-3p and Bax. For the luciferase assay, we used inhibitor, mimics and negative control. The mutant (mut) and wild-type (wt) 3′UTR region of Bax was amplified and was cloned in the pmiR reporter luciferase vector. The Phusion Site-Directed Mutagenesis Kit (ThermoFisher, USA) was used for the site-directed mutagenesis of Bax 3′UTR in the potential binding site of miR-142-3p. The BV-2 cells (2 × 10^6^ cells/well) were incubated in 96-well plates followed by co-transfection with 3′UTR-pMIR-Bax or 3′UTR-pMIR-Bax-mut, miR-142-3p inhibitor, mimics and respective negative control using Lipofectamine-2000 reagent.

### 2.10 Western blot analysis

The spinal cord tissue portion at the lesion site was isolated followed by homogenization, and total proteins were isolated. The BV-2 cells were also processed to isolate proteins. The samples were centrifuged (10,000*g*) at 4℃ for 30 min; the supernatant was subjected to the bicinchoninic acid assay for studying the protein expression levels. The proteins (30 µg) were subjected to electrophoresis followed by the transfer to Millipore PVDF membranes. The membranes were blocked by nonfat milk (5%) at 4℃ for 12 h. The membranes were then incubated with I^ry^ antibodies Bax (1:1,000), Bcl-2 (1:1,000), cleaved-caspase-9 (1:1,000) and pro-caspase-3 (1:1,000), and Actin was opted as a loading control. The membranes were incubated along with anti-mouse IgG horse radish peroxidase-conjugated II^ry^ antibody (1:200) for 60 min. The proteins were detected by quantifying the protein bands with the help of ImageQuant TL 8.2 image analysis software (GE health care and life sciences, USA).

### 2.11 Immunohistochemistry studies

For immunohistochemistry, the spinal tissues were subjected to intracardiac perfusion using isotonic sodium chloride solution and then by PFA (4%) in 0.1 M PBS at 4℃ for 20 min. Subsequently, a 10 mm portion of the spinal cord tissue at the injured site was isolated and was fixed in a paraffin block, and 5 µm thick section was prepared using a microtome. The paraffinized sections were treated with xylene for deparaffinization at room temperature for 2 min and hydrated using alcohol. The sections were incubated for 15 min in H_2_O_2_ (3%) at room temperature, followed by washing by 0.01 PBS. The sections were then blocked with fetal bovine serum (10%) in PBS for 20 min at room temperature and incubated at 4℃ for 12 h with anticleaved caspase-3 antibodies (1:1,000). Then, the sections were incubated along with anti-mouse IgG horseradish peroxidase II^ry^ antibodies (1:1,000) at room temperature for 20 min. Finally, 3,3′-diaminobenzidine staining was done to study the immunoreactivity. The images were viewed under the Olympus light microscope (Olympus Corp.). The images were quantified using Image scope-9 software.

### 2.12 Flow cytometry analysis for apoptosis

The BV-2 cells were washed using PBS (ice cold) and were then fixed in ethanol (70%, ice-cold) in PBS for 20 min. We used the FITC Annexin V Apoptosis detection kit for evaluating apoptosis. The cells were washed using PBS and were then resuspended in Annexin V buffer and incubated in Annexin V-FITC and propidium iodide. Cells receiving staining were evaluated with the flow cytometer (BD Biosciences, USA).

### 2.13 Evaluation of caspase-3 activity

The activity of caspase-3 was evaluated by the colorimetric assay kit following the supplied instructions. The BV-2 cells isolated by centrifugation at 5,000 rpm for 5 min at 4℃ and were incubated in lysis buffer for 10 min. Then, the lysates were subjected to centrifugation (10,000*g* for 20 min) at 4℃. The lysates were screened for protein levels using the protein estimation kit. The lysates were incubated with 0.2 mM DEVD-pNA buffer at room temperature. The samples were analyzed using a microplate reader at 405 nm.

### 2.14 Statistics

The statistics was done using Graphpad Prism software. All the experiments were done in triplicate, and the results are presented as mean ± standard deviation. Spearman’s rank correlation coefficient was done for analyzing the difference between Bax and miR-142-3p. One-way ANOVA along with Tukey’s *post hoc* analysis was done for establishing the differences between groups. Student’s *t*-test was done to establish differences between two groups. *P* < 0.05 was considered as statistically significant.

## 3 Results

### 3.1 Aberrant expression of miRs after SCI

**Figure 1:**
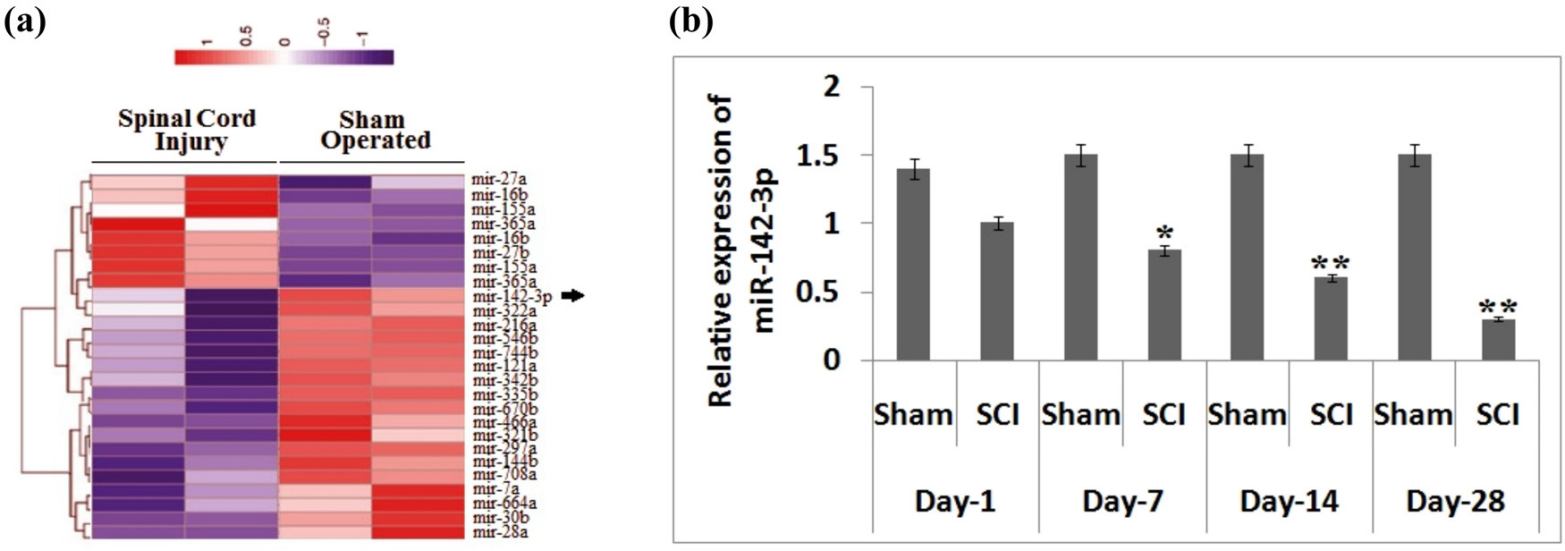
Expression analysis of miR expression in rats subjected to SCI. (a) The results of the heat map analysis showing changes in expression levels of miRNA at the end of the 14th day after submitting them to SCI. Violet color shows downregulation, and red color shows overexpression of miRs. (b) Quantitative results of RT-PCR for showing the expression of miR-142-3p in spinal tissues after SCI at day 1, day 7, day 14 and day 28 after SCI. The results are presented as mean ± SD. *P < 0.05, **P < 0.01 compared to sham-operated rats.

To study the possible involvement of miRs in spinal injury, we created an animal model of SCI followed by the microarray analysis for identifying the expression of miRs in spinal tissues. We observed multiple miRs showing altered expression at 14 days after SCI, and of them, miR-142-3p was the most prominently downregulated compared with sham-operated rats (Figure 1a). Previously, miR-142-3p has been found to be overexpressed in dorsal root ganglion (DRG) neurons [23]. Hence, we performed qRT-PCR to further confirm the expression levels of miR-142-3p in the spinal cord tissues on day 1, day 7, day 14 and day 28 after SCI. We observed that the levels of miR-142-3p were suppressed significantly (*P* < 005) in the spinal cord-injured group of rats compared to that in the sham group between the 7th and the 28th day (Figure 1b). These findings confirmed that SCI leads to aberrant levels of miRs in the spinal tissues and the possible involvement of miR-142-3p in the SCI.

### 3.2 Upregulation of miR-142-3p attenuates functional recovery and inhibits apoptosis after SCI.

**Figure 2:**
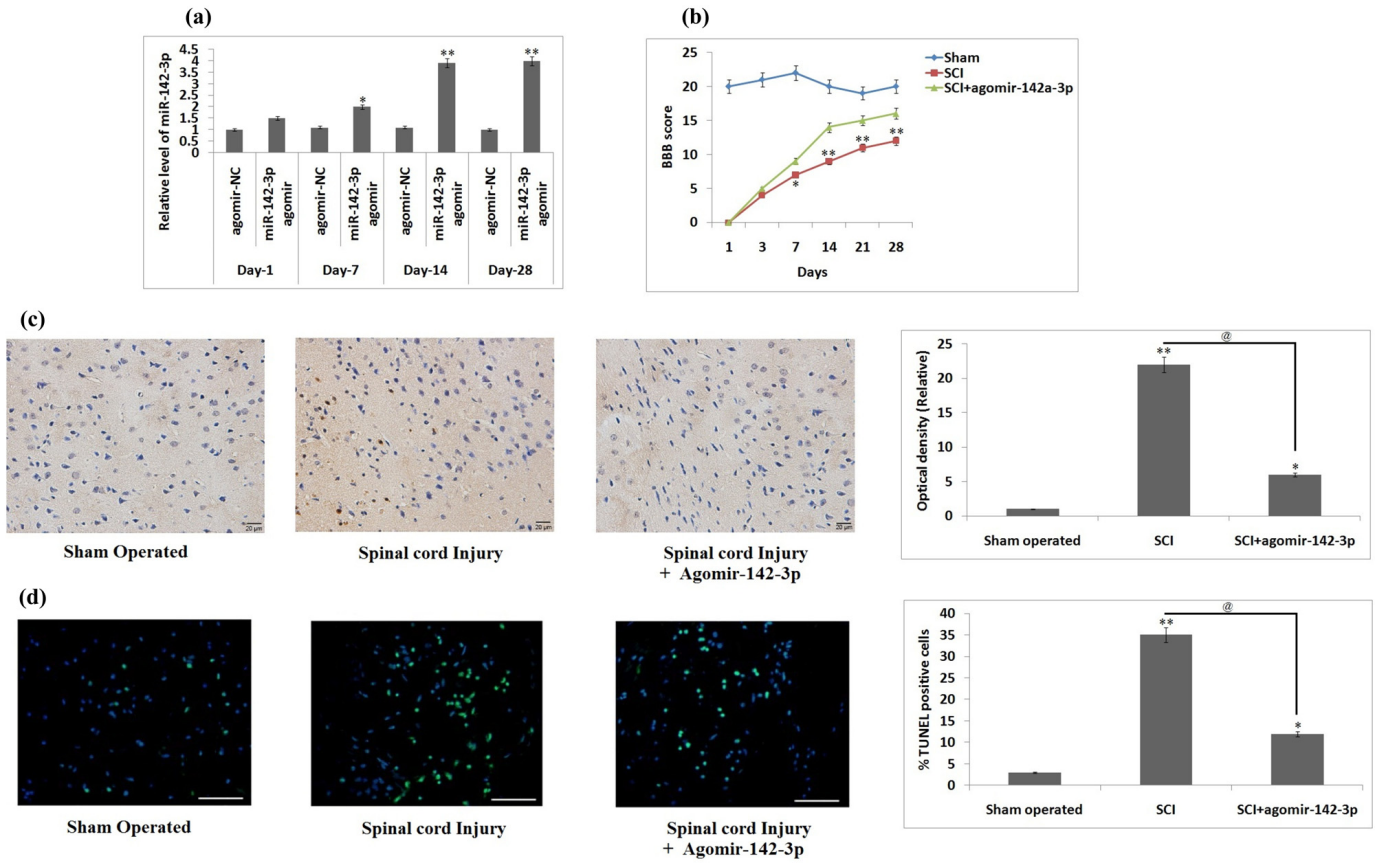
miR-142-3p agomir increases SCI. (a) Expression of miR-142-3p relative to agomir-NC detected at various days in rats submitted to spinal injury following the dose of miR-142-3p agomir. *P < 0.05, **P < 0.01 compared to agomiR-NC. (b) Results of BBB score for assessing the locomotor activity at various time intervals after spinal injury following the treatment of mir-142-3p. (c) Immunohistochemistry analysis for detecting the expression of cleaved-caspase-3 in the spinal tissues after SCI (magnification at 200×). *P < 0.05, **P < 0.01 compared to sham-operated rats, *P* < 0.05 compared to spinal injury rats. (d) Results of the TUNEL assay for identifying extent of apoptosis of neuronal cells on the 14th day after spinal injury (magnification at 200×). The results are presented as mean ± SD.

To find the role of miR-142-3p in the rats subjected to the SCI, we developed a rat model and treated the rats with miR-142-3p agomir via the intrathecal route. qRT-PCR was done to evaluate the effect of overexpression by miR-142-3p agomir in the spinal tissues. We found that the relative levels of miR-142-3p were significantly overexpressed (Figure 2a) between the 7th and the 28th day compared to the rats treated with agomir-NC (*P* < 0.05) with the maximum levels on the 14th day. Furthermore, in our study, we utilized the BBB rating score for evaluating the motor function in the spinal cord-injured rats after treating them with miR-142-3p agomir. The results suggested that the upregulation of miR-142-3p in the SCI + miR-142-3p agomir group of rats showed a significant improvement in the motor function form the 7th day against the SCI rats (*P* < 0.05, Figure 2b). Subsequently, the results of Cresyl staining suggested that the SCI + miR-142-3p agomir-treated SCI rats showed an increased amount of the spared tissue compared to the SCI rats, suggesting that miR-142-3p agomir decreased the lesion volume in spinal tissues after SCI. In addition, we studied whether miR-142-3p regulates the expression of apoptosis-involved proteins such as cleaved-caspase-3 by immunohistochemistry analysis in the spinal cord tissues after injury. As shown in Figure 2c, SCI caused a significant overexpression of cleaved-caspase-3 in the spinal cord tissues against the sham group, whereas the treatment of miR-142-3p agomir resulted in the inhibition of cleaved-caspase-3 (*P* < 0.05, Figure 2c). In addition, the neuronal cell apoptosis was evaluated by the TUNEL assay. It was found that the number of TUNEL-positive cells increased significantly (*P* < 0.01) in the SCI rats compared to the sham-operated group; however, miR-142-3p agomir significantly decreased the TUNEL-positive cells in the SCI rats treated with miR-142-3p agomir against the SCI rats (*P* < 0.01, Figure 2d). These findings suggest that miR-142-3p agomir improved the functional recovery, suppressed apoptosis and decreased the lesion volume in rats after SCI.

### 3.3 miR-142-3p causes downregulation of Bax via targeting the 3′UTR in BV-2 cells

**Figure 3:**
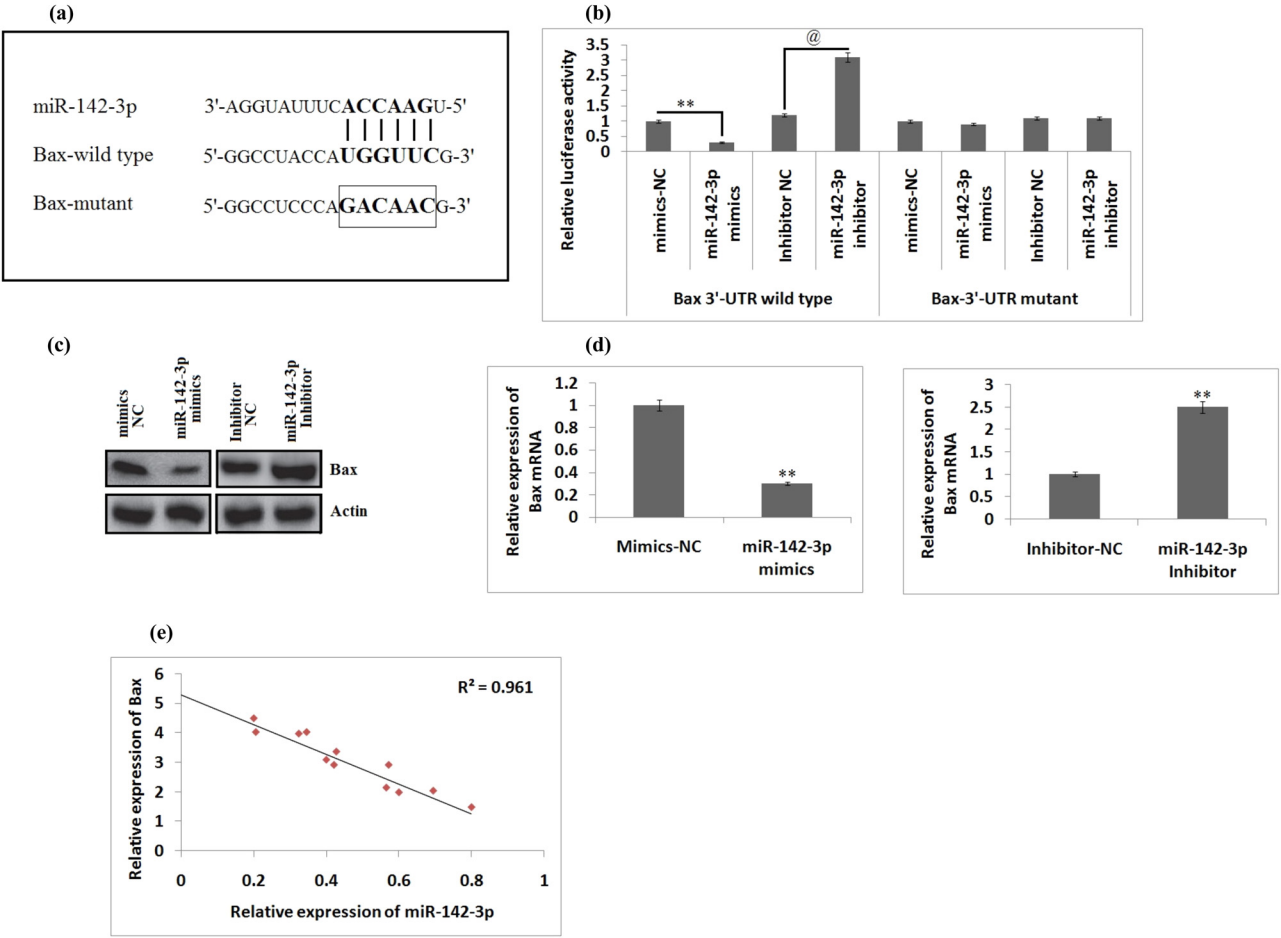
Bax is the potential target of miR-142-3p in BV-2 cells. (a) TargetScan predicted potential binding sites on the 3′UTR of Bax mRNA. (b) Quantitative results of luciferase activity of Bax-wt or mut 3′UTR in cells transfected with miR-142-3p mimics/inhibitor and NC. **P < 0.01, and *P* < 0.01 compared to NC. (c) Protein expression by western blot analysis. (d) Quantitative results of mRNA expression for Bax done by western blot analysis and qRT-PCR. **P < 0.01 compared to a negative control. (e) Quantitative results showing a negative correlation between expression of miR-142-3p and Bax (*r* = 0.961). The results are presented as mean ± SD.

Previously, it has been reported that miR-142-3p inhibits apoptosis and promotes the neuronal cell cycle [23], and in addition, a report confirmed the involvement of miR-142 in apoptosis via downregulation of pro-apoptosis proteins caspase-3 and Bax [25]. Therefore, we speculated that miR-142-3p inhibits the apoptosis in neuronal cells of rats subjected to the SCI via suppressing the levels of Bax. Subsequently, the bioinformatics analysis was done to predict the potential targets of miR-142-3p, and it was noticed that Bax may be a potential target of miR-142-3p having the potential binding site in the 3′UTR (Figure 3a). We further validated the bioinformatics data, and the Bax-3′-UTR constructs (wild type (wt) and mutant (mut)) were inserted in the firefly luciferase-expressing vector pmiR-reporter. To evaluate the pathologic parameters after SCI, we used the BV-2 cells, which are reported to have various features of I^ry^ microglia [25]. The plasmids received co-transfection of mir-142-3p mimics/inhibitor or NC mimics/inhibitor in the BV-2 cells followed by evaluation of luciferase activity. We observed that miR-142-3p-mimics caused the inhibition of the luciferase activity significantly compared to NC mimics in the presence of 3′UTR (wt), whereas the inhibitor resulted in the increased luciferase activity against the inhibitor (NC; *P* < 0.01, Figure 3b). In addition, miR-142-3p did not suppress the activity of luciferase in the vector bearing 3′-UTR of Bax having mutations in the miR-142-3p-binding site (Figure 3b).

To further evaluate whether Bax is regulated negatively by miR-142-3p, we performed western blot and qRT-PCR analyses to determine protein and mRNA levels, respectively. It was noticed that upregulated miR-142-3p decreased the levels of Bax as both protein and mRNA levels in BV-2 cells (Figure 3c and d). Furthermore, the RT-PCR study was performed to find the mRNA levels of Bax in the spinal cord tissue, and we evidenced that the levels of mRNA of Bax were significantly overexpressed in the spinal cord tissue compared to that in sham-operated rats (*P* < 0.01, Figure 3d). The overall analysis suggested a significant negative correlation between miR-142-3p and Bax in the spinal cord tissue (Figure 3e). Overall, the findings of the experiment established that miR-142-3p suppressed the levels of Bax via targeting the 3′UTR site in BV-2 cells, hence suggesting Bax as a possible target of miR-142-3p in spinal cord tissues.

### 3.4 Upregulation of Bax blocks the protective activity of miR-142-3p in hydrogen peroxide-exposed BV-2 cells

**Figure 4:**
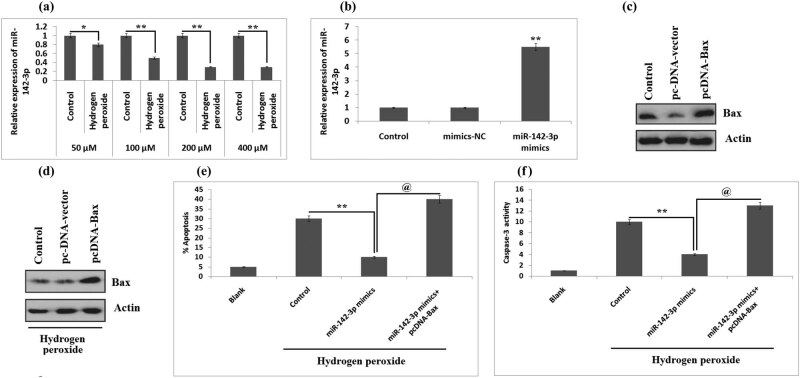
Restitution of Bax blocks the protective effect of miR-142-3p in hydrogen peroxide-exposed BV-2 cells. (a) The BV-2 cells were exposed to hydrogen peroxide of varied concentrations (50–400 μM) for 10 h followed by qRT-PCR evaluation of miR-142-3p expression. *P < 0.05, **P < 0.01 compared to the control group of cells. (b) qRT-PCR analysis for expression of miR-142-3p in BV-2 cells after transfection with miR-142-3p mimics or NC. **P < 0.01 compared to NC mimics. (c) BV-2 cells received transfection of pc-DNA-Bax or pc-DNA-vector followed by western blot analysis for expression of Bax. (d) Results of western blot analysis for expression of Bax in hydrogen peroxide-treated cells, which received transfection of miR-142-3p mimics or miR-142-3p and pc-DNA-Bax. After treatment with hydrogen peroxide, the cells were transfected with miR-142-3p-mimics or miR-142-3p-mimics and pc-DNA-Bax. (e) Quantitative results of % apoptotic cells. (f) Quantitative results of capase-3 activity. **P < 0.01, @*P* < 0.01. The results are presented as mean ± SD.

Studies earlier have demonstrated that reactive oxygen species play an important role in the SCI as they activate multiple pathways of apoptosis, and hydrogen peroxide-exposed BV-2 cells are established as a cellular model of SCI for evaluating the pathologic factors after the SCI [26]. In the present work, we used murine BV-2 cell lines that were exposed with hydrogen peroxide of varied concentrations (50–400 µM) for 10 h after which the levels of miR-142-3p were studied using RT-PCR. The results (Figure 4a) suggested that hydrogen peroxide caused a significant downregulation of miR-142-3p in BV-2 cells in a concentration-dependent manner between concentrations 50 and 200 µM (*P* < 0.05). Subsequently, western blot and RT-PCR analyses were carried out to study the upregulation of miR-142-3p or Bax. The outcomes suggested that miR-142-3p and Bax were overexpressed in Bv-2 cells treated with miR-142-3p mimics pc-DNA-Bax (Figure 4b and c). Also, it was found that the upregulation of miR-142-3p downregulated the protein expression of hydrogen peroxide-induced BV-2 cells, whereas the pc-DNA-Bax inhibited the restoration of Bax. miR-142-3p reduced the expression of Bax in hydrogen peroxide-treated cells (Figure 4d). The results of the present experiment suggested that the upregulation of miR-142-3p suppressed the number of apoptotic cells in hydrogen peroxide-exposed Bv-2 cells against the control cells significantly, but the protective effect of miR-142-3p decreased significantly with the overexpression of Bax (Figure 4e, *P* < 0.01). Furthermore, the overexpression of Bax halted the effect of miR-142-3p-blocked caspase-3 activity in hydrogen peroxide-treated cells (*P* < 0.01, Figure 4f). Taken together, the outcomes suggested that miR-142-3p inhibits apoptosis of cells via inhibiting Bax in hydrogen peroxide-treated Bv-2 cells.

### 3.5 Upregualtion of miR-142-3p inhibits the mitochondrial apoptosis cascade

**Figure 5:**
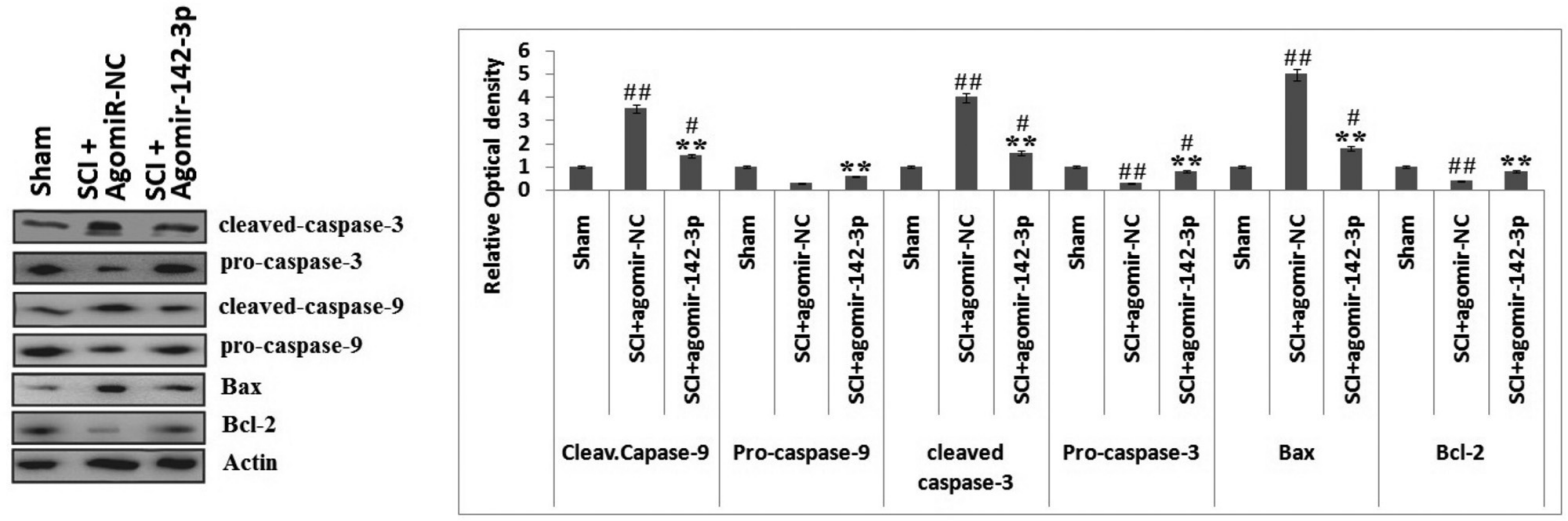
miR-142-3p inhibits the mitochondrial apoptotic pathway. The rats submitted to spinal injury followed by the treatment of miR-142-3p agomir, agomir-NC followed by western blot analysis for expression of calaved-caspase-9/-3, pro-caspase-9/-3, Bax, Bcl-2 in the spinal injured tissue. The results are presented as mean ± SD. **P < 0.01 compared to the spinal cord-injured + agomir NC rats. ##*P* < 0.01, #*P* < 0.05 compared to sham-operated rats.

Bax, a protein belonging to Bcl-2 family, has been reported to show the pro-apoptotic effect and also leads to the release of CYT-*C* [27,28,29]. In the process of apoptosis, the overexpression of Bax may encourage apoptosis via blocking Bcl-2, which is an antiapoptotic protein [30]. It had been reported earlier that both the death receptor and the mitochondrial apoptosis pathway (MAP) are the important pathways [31,32]. Bax is an important regulator in the MAP, and the molecule is able to build up homodimers on the mitochondrial membranes and lead to the opening of permeability pores on it for releasing CYT-*C* from the mitochondria in the cytoplasm [33]. In the MAP, CYT-*C* may activate the caspase-9-induced reaction, which converts pro-caspase-3 to release caspase-3 [34]. To find whether miR-142-3p is involved in the regulation of MAP by inhibiting the levels of apoptosis-associated protein in rats post SCI, the immunoblotting analysis was performed to assess the expression levels of pro-caspase-9, cleaved-caspase-9, pro-caspase-3, cleaved-caspase-3, Bcl-2 and Bax in the isolated spinal cord tissues. It was noticed that the expressions of cleaved-caspase-3/-9 and Bax were overexpressed significantly, and the levels of pro‑caspase‑3/-9 and Bcl-2 were found to be downregulated in the SCI + NC agomiR group against the sham-operated group. However, the upregulation of miR-142-3p significantly decreased the expression of cleaved-caspase-3/-9 and Bax and significantly elevated the expression of pro-caspase-3/-9 and Bcl-2 in SCI + miR-142-3p agomir group against the SCI + NC-agomiR group (*P* < 0.01, Figure 5). These findings suggested that the upregulation of miR-142-3p can inhibit the MAP via suppressing the levels of Bax in rats after SCI.

## 4 Discussion

The SCI causes a number of biochemical and molecular changes in the body, which are associated with the production of free radicals, triggering inflammatory response, neuronal cell death and plasticity of axions [35,36]. Previous studies have established that SCI can lead to aberrant expression of miRNA, and deregulated miRs can affect the pathophysiology of SCI and its functional outcomes [37]. However, studies confirming the specific role of miRs in the SCI are missing. In the present work, an animal model of SCI was developed, and microarray analysis was done to screen the expression profiles of miRs after SCI. It was noticed that SCI caused deregulation in the expression of miRs, among which miR-142-3p was one of the most significantly deregulated in the spinal tissues. In addition, it was evidenced that the upregulation of miR-142-3p improved the SCI by attenuating functional recovery, decreasing the size of lesions and apoptosis. Furthermore, it was found that miR-142-3p inhibited the levels of Bax via 3′UTR region in the BV-2 cells exposed to hydrogen peroxide. However, the present outcomes suggested that miR-142-3p shows its protective action on the SCI via inhibiting the MAP.

miR-142-3p is identified to play an important role in inflammation, viral infection, cancer, and immunity [38]. miR-142-3p is associated to be linked with neuronal disorders, and it has also been identified to be a key target in repairing of sensory function in neurons through adenylyl cyclase [39]. miR-142-3p miR-142-3p is responsible for regulating the proinflammatory function in lupus erythematosus [40]. miR-142-3p has also been found to be overexpressed in the rat dorsal route ganglion after sciatic nerve transection in the first 24 h, and in addition, it was also identified in apoptosis and cell growth after sciatic injury [23]. miR-142-3p has also been reported to play an important therapeutic target in SCI [39]. Although these studies have established the role of miR-142-3p in neuronal development, sciatic nerve injury and functional recovery in SCI, its specific role and the involved pathway in SCI remain unclear. Here, we studied the expression profile of miRs in the animal model of SCI in the post-injury phase. We evidenced that miR-142-3p was the most downregulated miR, which was consistent with the findings of the earlier study [39].

Mitochondria play a significant role in the process of apoptosis by releasing the molecules responsible for apoptosis such as CYT-*C* [41]. Bax also called as BCL2-associated X protein is a pro-apoptotic protein, which can open the transition pores present on the mitochondria membrane, facilitating the release of CYT-*C* from the mitochondria [33]. CYT-*C* can promote the amplification reaction of the caspase‑9‑molulated pathway in the MAP, which in turn forces pro-caspase-3 to form caspase-3 [34]. Here, it was evidenced that miR-142-3p decreases the levels of Bax via targeting the 3′UTR region in the BV-2 cells. In addition, our results suggested a negative link between expression of miR-142-3p and Bax in the spinal cord tissues of the spinal cord-injured rats, suggesting Bax as a favorable target of miR-142-3p *in vivo.* Hence, we speculated that miR-142-3p may modulate the MAP via blocking the expression of Bax in rats subjected to SCI. The present findings suggested that the increased expression of miR-142-3p suppressed the cleaved-caspase-3/-9 and Bax and increased the expression of pro-caspase-3/-9 and Bcl-2 in the spinal cord tissues of SCI-induced rats. These findings suggested that miR-142-3p may show its therapeutic effect in SCI via blocking the MAP. However, the present study has some shortfalls, i.e., the study focused on only the most overexpressed miR-142-3p, and we do not screen other miRs for their role in SCI.

In conclusion, the present work showed that SCI leads to aberrant expression of miRs in the rats subjected to SCI, and among miRs, miR-142-3p was the most significantly decreased miR in the spinal cord. Also, it was evidenced that the increased levels of miR-142-3p improved the functional recovery, decreased the lesion size and also suppressed the apoptosis of neuronal cells post-SCI. This work also confirmed that miR-142-3p targets Bax in the BV-2 cells and may show its protective effect in SCI via inhibiting the MAP, suggesting miR-142-3p can be an important therapeutic target for treating SCI.
